# Influence of Calcium Phosphate and Apatite Containing Products on Enamel Erosion

**DOI:** 10.1155/2016/7959273

**Published:** 2016-06-26

**Authors:** A. Kensche, S. Pötschke, C. Hannig, G. Richter, W. Hoth-Hannig, M. Hannig

**Affiliations:** ^1^Clinic of Operative and Pediatric Dentistry, Medical Faculty Carl Gustav Carus, TU Dresden, Fetscherstraße 74, 01307 Dresden, Germany; ^2^Clinic of Prosthetic Dentistry, Medical Faculty Carl Gustav Carus, TU Dresden, Fetscherstraße 74, 01307 Dresden, Germany; ^3^Clinic of Operative Dentistry, Periodontology and Preventive Dentistry, University Hospital, Saarland University, Building 73, Saarland, 66421 Homburg, Germany

## Abstract

For the purpose of erosion prevention the present study aimed to compare the efficacy of two biomimetic products and a fluoride solution to optimize the protective properties of the pellicle. After 1 min of* in situ* pellicle formation on bovine enamel slabs, 8 subjects adopted CPP-ACP (GC Tooth Mousse), a mouthwash with hydroxyapatite microclusters (Biorepair), or a fluoride based mouthwash (elmex Kariesschutz) for 1 min each. Afterwards, samples were exposed in the oral cavity for 28 min. Native enamel slabs and slabs exposed to the oral cavity for 30 min without any rinse served as controls. After oral exposure, slabs were incubated in HCl (pH values 2, 2.3, and 3) for 120 s and kinetics of calcium and phosphate release were measured photometrically; representative samples were evaluated by SEM and TEM. The physiological pellicle reduced demineralization at all pH values; the protective effect was enhanced by fluoride. The biomimetic materials also reduced ion release but their effect was less pronounced. SEM indicated no layer formation after use of the different products. However, TEM confirmed the potential accumulation of mineral components at the pellicle surface. The tested products improve the protective properties of the* in situ* pellicle but not as effectively as fluorides.

## 1. Introduction

Caries and erosion are both attributed to demineralization processes at the dental hard tissues. However, due to the crucial differences regarding their etiology, pathogenesis, and morphological manifestation, different preventive approaches are required [1–3]. Either way, interactions with salivary components as well as with the pellicle layer considerably contribute to the mode of action of different preventive agents [3–5]. Since the proteinaceous pellicle layer is omnipresent on all solid surfaces exposed in the oral cavity, inevitably functional active ingredients interact primarily with the pellicle components before reaching the dental tissue [5]. Due to the pellicle's network-like structure it can be suggested that particles can diffuse to the enamel surface.

Fluoridated tooth pastes and mouthwashes are by far the most frequently used preparations in oral hygiene measures [6–8]. Besides the release of antibacterial effects, the regular application of fluoride ions to a certain extend promotes the formation of fluorohydroxyapatite which is valued for its decreased solubility [1]. Furthermore, it is generally accepted that the accumulation of sodium or amino fluoride at the enamel surface leads to the precipitation of calcium fluoride-like deposits which subsequently release fluoride over time [1, 2]. In this context it has been suggested that a modification of the protein composition of the pellicle might enhance this effect [4]. While these advantages are undisputable regarding the prevention and remineralization of initial carious defects, their efficacy in order to decrease erosion associated enamel dissolution leaves room for improvement [9]. In case of strong or frequent erosive attack the potential calcium fluoride layer appears to be not dense or resistant enough to prevent enamel dissolution but may at most serve as a limited fluoride reservoir [1, 6].

The abundance of recent literature confirms that nano- and microparticle based prophylactic strategies gain great attention in scientific dental research [5, 10–12]. Modern biomimetic nanomaterials seem to provide alternative approaches for the prevention of mineral loss and for remineralization of initial enamel lesions [11, 13]. Some preparations try to mimic the smallest building units of dental enamel, the hydroxyapatite microcrystallites [11]. These biomimetic particles are assumed to accumulate at the tooth surface with high affinity and to regenerate porous initial lesions [14–16]. The efficacy of toothpastes and mouthwashes containing hydroxyapatite microclusters in promoting the deposition of a carbonate-hydroxyapatite particle coating on eroded enamel has already been under investigation* in vitro* [17, 18]. However, uncertainty remains about the sustainability of such mineral deposits and there is sparse evidence for the efficacy of this approach* in vivo* or* in situ* [19]. Furthermore it has been suggested that the addition of hydroxyapatite nanoparticles to an erosive soft drink significantly decreases its erosive potential by providing a calcium and phosphate supersaturation [20].

A more widespread and intensively examined approach is the application of CPP-ACP (casein phosphopeptide-amorphous calcium phosphate) nanocomplexes [21–23]. Due to their multiple phosphoryl residues, casein phosphopeptides have the ability to bind to clusters of amorphous calcium phosphate, stabilizing them in a metastable solution [24]. Numerous studies indicate that, if applied to the surface of dental hard tissues, CPP-ACP serves as a reservoir of bioavailable calcium and phosphate preventing sustained demineralization [25–27]. Some* in vitro* studies have been performed involving scanning electron microscopy and atomic force microscopy to analyze the structural alteration of dental hard tissue after erosive attack under the influence of CPP-ACP application [15, 23]. According to their results, CPP-ACP application before, between, or after acid exposures could decrease acid derived surface roughness significantly, possibly due to an ion deposition rather than crystal regrowth [23]. Wang et al. also hypothesized an erosion-preventive effect. However, determination of the enamel surface nanohardness before and after prophylactic treatment and erosive attack did not confirm this potential effect [28]. According to White et al. [29], the presence of casein phosphopeptides might also have a relevant buffering effect as the proteins retain proton-concentration from the enamel surface. Nevertheless, an* in vitro* approach as conducted in most studies disregards the possible interaction of CPP-ACP with pellicle components and neglects the influence of salivary dilution [30]. The* in vitro* determination of calcium and phosphate loss from enamel samples in an acidic milieu after previous exposure* in situ* with and without application of certain oral healthcare preparations allows the quantification of mineral loss during short time erosive attacks in an* in situ*/*in vitro* model [31–34]. The aim of the present study was to compare the effect of a conventional fluoride preparation and of modern calcium phosphate or apatite containing prophylactic products on initial erosive mineral loss in a combined* in situ*/*in vitro* setup. Surface alterations and layer formation were visualized electron-microscopically.

## 2. Materials and Methods

### 2.1. Subjects

The study was carried out with the support of 8 healthy volunteers who were all members of the laboratory staff (age 24–42). Based on the visual examination by an experienced dentist, the subjects all presented a good oral health with no signs of gingivitis, caries, or unphysiological salivary flow rate.

Beforehand, the study design was reviewed and approved by the Ethics Committees of the Saarland University (# 18/10) and of the Medical Faculty, TU Dresden (EK 147052013), and all volunteers had given their informed written consent about participation in the study.

### 2.2. Preparation of Specimens

The required cylindrical enamel slabs (5 mm diameter; 19,63 mm^2^ surface area and 1 mm thickness) were gained from the bovine incisor teeth of 2-year-old cattle. As described previously, the specimens were subjected to 37% phosphoric acid gel for 15 s on all surfaces with the exception of the outer enamel surface (Etching gel, DMG, Hamburg, Germany) and pretreated for 30 s with Optibond Primer (Kerr, Karlsruhe, Germany) [34] before Optibond Adhesive was used and light-cured for 30 s. Subsequently, the unsealed enamel surfaces were wet-ground and polished in a standardized grinding procedure with up to 4000-grid abrasive paper [3, 32] until approximately 200 *μ*m of the enamel was removed. In case of structural enamel alterations specimens were excluded from the study. The removal of the smear layer was achieved by steam jet and ultrasonication (US) with 3% NaOCl for 3 min [32, 35]. After being washed twice in distilled water for 5 min by US, the samples were disinfected in ethanol (70%) for another 10 min (US), washed again, and finally stored in distilled water for 24 h before the* in situ* experiments [36].

### 2.3. *In Situ* Pellicle Formation and* In Vitro* Erosion

**Figure 1:**
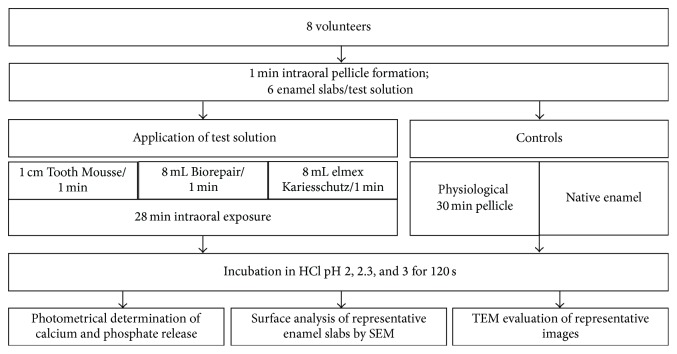
Flowchart of the experiments.

*In situ* pellicle formation was carried out with individual upper jaw splints. Since all* in vitro* experiments (pH values 2, 2.3, and 3) were conducted in duplicate measurements, 6 enamel slabs were exposed intraorally at a time [33]. Therefore, cavities were prepared in the buccal aspects of the splints (regions 16, 15, and 14, regions 24, 25, and 26, resp.) and the samples were placed on each splint with polyvinyl siloxane impression material (President Light Body, Coltene, Switzerland) so that only the specimens' surfaces were exposed to the saliva. Intraoral exposure was generally performed between 7 and 12 a.m. All participants were instructed to brush their teeth without toothpaste and to then avoid any intake of food or drinks other than water for 2 hours before the scheduled beginning of the* in situ* experiments. The flowchart in Figure 1 outlines all the* in situ* as well as* in vitro* experiments performed in this study.

The splints were carried intraorally for 1 min to allow initial pellicle formation on the surfaces before the subjects adopted the different preparations as follows: either they rinsed thoroughly with 8 mL of the different mouthwashes (Biorepair, Dr. Wolff, Bielefeld, Germany, or elmex Kariesschutz, GABA, Lörrach, Germany) for 1 min or the CPP-ACP compound was applied for 1 min with the fingertip and 1 cm of the paste was adopted (GC Tooth Mousse, GC Europe, Leuven, Belgium). The main ingredients contained in the prophylactic agents are enlisted in Table 1. The splints remained in the oral cavity for further 28 min; this regime gave a total intraoral exposure time of 30 min.

After intraoral exposure, the slabs were quickly removed from the splints and thoroughly rinsed with running water for 5 s. In accordance with previous studies, hydrochloric acid (pH values 2, 2.3, and 3) served as the erosive agent. The enamel slabs were incubated in 1000 *μ*L of the solution* in vitro* to provide an excess of acid in order to prevent pH variations during incubation for 120 s [31, 33, 34]. Before exposure to HCl the enamel slabs were embedded with silicone impression material at the bottom of a 2 mL Eppendorf cup. A constant circulation of the solution was achieved by continuous pumping with a 100 *μ*L pipette (1 lift/s); every 15 s 100 *μ*L of the acid was removed for photometric analysis and replaced straight away by 100 *μ*L of fresh acid. Also, enamel specimens were coated by a 30 min salivary pellicle without rinses, and slabs without intraoral exposure served as controls.

To avoid mutual interference of the preparations, experiments with different materials and control experiments were carried out on different days.

In this study, the demineralization induced by incubating the enamel samples in HCl was determined on the basis of photometrically quantified calcium and phosphate release into the solution. Therefore, a double assay using the Arsenazo III method (Fluitest®, Ca-A-II, analyticon, Lichtenfels, Germany) and the malachite green assay [34, 37, 38] was performed.

The interaction of Arsenazo III with calcium in an acid solution results in the formation of a blue purple complex with its intensity developing proportionally to the calcium concentration. Absorption is determined at *λ* = 650 nm according to standard curves. The working solution used in this assay was composed of 100 mmol/L imidazole buffer (pH 6.5) and 0.12 mmol/L Arsenazo III. For each measurement a volume of 10 *μ*L from the sample was added to 100 *μ*L Arsenazo reagent [34], always performed as a duplicate test, with the average adsorption being calculated.

Similarly, malachite green reacts with phosphate to give a colored complex which exhibits its adsorption maximum at *λ* = 650 nm. As previously described, for the test reagent 0.045 mg of malachite green dissolved in 100 mL aqua bidistilled water was mixed with 12.69 g ammonium molybdate dissolved in 300 mL HCl (4 mol/L). The reagent was stirred for 30 min afterwards and filtered (pore size 0.22 *μ*m) [34].

A volume of 10 *μ*L from the sample was pipetted to 200 *μ*L malachite-reagent; the absorption was measured after 15 min [34]. Again, two measurements were performed for each specimen and the average absorption was calculated.

### 2.4. Electron Microscopic Evaluation

In addition to the photometrical determination of the acid-induced calcium and phosphate release, the direct impact of the prophylactic agents on the pellicle as well as on the enamel slabs was visualized by scanning and transmission electron microscopy, respectively.* In situ* experiments were carried out as described above and the specimens were incubated* in vitro* in HCl for 60 s. Afterwards surface analysis of the enamel slabs was performed under a XL 30 ESEM scanning electron microscope (Philips, Eindhoven, Netherlands) and representative images were taken at a 6,500-fold magnification.

Furthermore, slabs were prepared for TEM. Fixation of the specimens was performed in a 1.5% formaldehyde-2.5% glutaraldehyde solution for 2 h. After being washed 5 times in phosphate buffer, the specimens were postfixed in 1% osmium tetroxide for 2 h to enable visualization of organic structures. Then all samples were increasingly dehydrated in ethanol and embedded in Araldite M (Serva, Darmstadt, Germany). Prior to microscopic examination the specimens were decalcified by 0.1 M HCl and reembedded in Araldite. Ultrathin sections of the remaining pellicle were cut in series with an ultramicrotome (Ultracut E, Reichert, Bensheim, Germany). The ultrathin sections were mounted on pioloform coated copper grids (Plano, Wetzlar, Germany) and contrasted with uranyl acetate and lead citrate [33, 39]. In order to distinguish inorganic components integrated in the pellicle layer, some ultrathin sections were analyzed by TEM without contrasting. TEM investigation was performed in a TEM TECNAI 12 Biotwin (FEI, Eindhoven, Netherlands) at x98.000 magnification, at which representative images were taken.

### 2.5. Statistics

Data were evaluated by one-way ANOVA followed by the Scheffé-procedure in order to detect significant influences of pellicle formation and additionally applied preparations on calcium and phosphate loss during incubation in HCl. The statistical analysis was performed considering the data from the 24 enamel slabs from all 8 subjects of each experimental subgroup after 120 s of incubation in HCl for calculation of the means that were compared for significant differences. Normal distribution was checked using the Kolmogorov-Smirnov test; the software used was SPSS 20.0 (StatSoft, Hamburg, Germany). Level of significance was set at *p* < 0.05.

## 3. Results

### 3.1. Dissociation of Calcium and Phosphate

**Figure 2:**
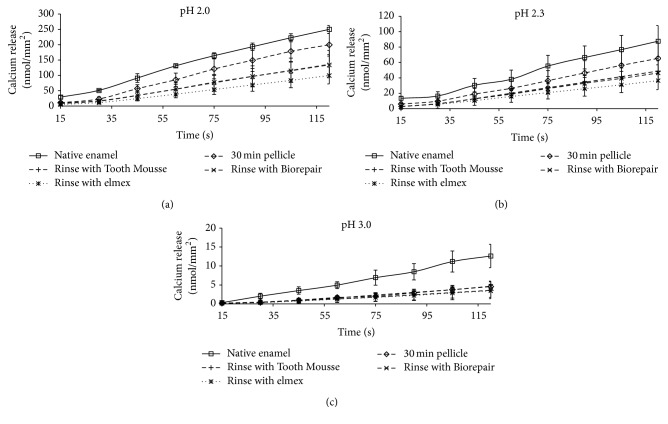
Kinetics of calcium loss during incubation of enamel slabs for 120 s with and without application of different preparations* in situ*. Physiological 30 min pellicles (without rinse) and specimens without pellicle served as controls. In summary the erosion-preventive effect of fluoride was confirmed and could not be achieved by the calcium phosphate and apatite based products. Please note the different scale; *n* = 8 subjects; *n* = 24 enamel samples per subgroup; mean values ± standard deviation.

**Figure 3:**
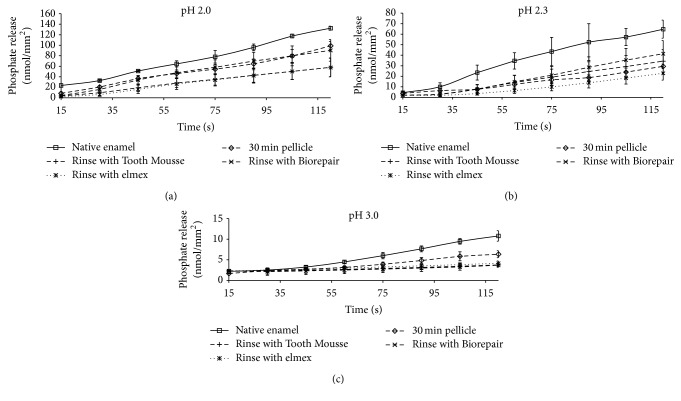
Kinetics of phosphate loss during incubation of enamel slabs for 120 s with and without application of different preparations* in situ*. Physiological 30 min pellicles (without rinse) and specimens without pellicle served as controls. On the whole, the phosphate release reducing effect of the calcium phosphate and apatite based products appeared inferior to fluoride. Please note the different scale; *n* = 8 subjects; *n* = 24 enamel samples per subgroup; mean values ± standard deviation.

First of all, the conducted measurements clearly demonstrate that the release of calcium as well as phosphate is linearly dependent on the corresponding pH values irrespective of the pretreatment of the enamel slabs. Yet, the formation of the 30 min pellicle reduced mineral loss as compared with bare enamel (Figures 2 and 3).

The additional application of elmex Kariesschutz, GC Tooth Mousse, or Biorepair mouth rinsing solution modified the calcium and phosphate loss; this effect was different at the tested pH values.

In all cases, preliminary application of elmex Kariesschutz enhanced the protective properties of the pellicle layer. During the 120 s incubation of the specimens in HCl the application of elmex Kariesschutz could decrease the calcium release by 73% at pH 3, 59% at pH 2.3, and 60% at pH 2 in comparison to the native enamel control. A similar effect could be confirmed for the phosphate release (61% at pH 3, 64% at pH 2.3, and 43% at pH 2).

GC Tooth Mousse and Biorepair mouth rinsing solution yielded similar efficacy at pH 3.0 with respect to the release of calcium and phosphate. However, at pH 2.3 Biorepair mouth rinsing solution and GC Tooth Mousse were less effective than elmex Kariesschutz considering the calcium release; the phosphate release at pH 2.3 was even higher than from samples coated with a physiological pellicle layer.

At pH 2.0, Biorepair mouth rinsing solution and GC Tooth Mousse were also less effective than elmex Kariesschutz with respect to the calcium release. A divergent observation was made for phosphate at pH 2.0: GC Tooth Mousse had the same effect as elmex Kariesschutz whereas Biorepair mouth washing solution was in line with the pellicle coated samples.

A significant impact of the different pretreatments on calcium release was observed at pH 2.3 (ANOVA *p* = 0.016) and pH 3.0 (*p* = 0.006). Additional pairwise comparison indicated significant differences of pellicle covered samples with the elmex group at pH 2.3 (Scheffé-procedure, *p* = 0.031) and at pH 3.0 (Biorepair versus native enamel, *p* = 0.013).

A significant impact of the preparations on phosphate release was observed at all pH levels (ANOVA *p* ≤ 0.001). Additional pairwise comparison (Scheffé-procedure) yielded significant difference of untreated controls and all other groups at pH 2 and pH 2.3 (pH 2: control versus pellicle, *p* = 0.005, control versus GC Tooth Mousse, *p* = 0.001, control versus Biorepair mouth rinsing solution, *p* = 0.012, and control versus elmex Kariesschutz, *p* = 0.001; pH 2.3: control versus all other groups, *p* ≤ 0.001). At pH 3 phosphate release from pellicle coated samples differed significantly from controls (*p* = 0.01), GC Tooth Mousse (*p* = 0.01), and from Biorepair mouth rinsing solution (*p* = 0.05).

### 3.2. Scanning Electron Microscopy

**Figure 4:**
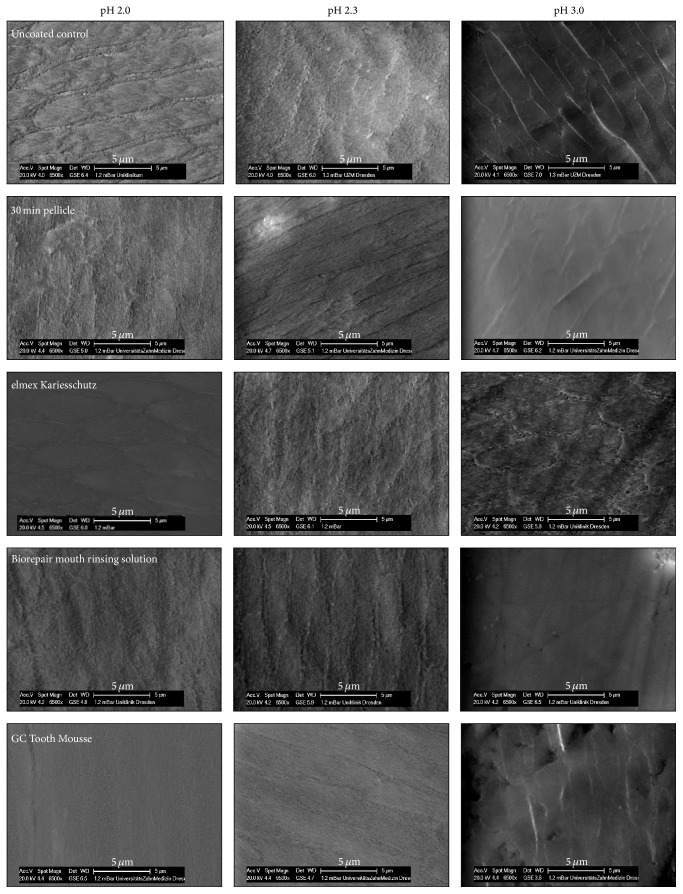
Incubation of enamel slabs in HCl for 60 s after pretreatment with different products* in situ*, scanning electron microscopic evaluation.

Scanning electron microscopy allowed the visualization of changes of the enamel surface after incubation in HCl* in vitro* (Figure 4). Due to the demineralization process, the surface of most samples appeared roughened, more obviously recognizable after incubation at pH 2.3 and pH 2.0, respectively. The exposure of prismatic structures with distinct depressions in the periphery was noticeable on the native as well as on the 30 min pellicle covered enamel slabs after erosive attack. Despite its effect on calcium and phosphate release (see above), in terms of enamel surface alteration due to HCl incubation, the application of elmex Kariesschutz did not result in a visible impact when compared with the pellicle covered enamel slabs or the uncovered control. Moreover, the structure of the etched enamel after application of Biorepair mouth rinsing solution also resembled that of the pellicle covered samples. In contrast, enamel surfaces treated with GC Tooth Mousse revealed a smoother texture possibly less affected by HCl incubation; the etching effect on the enamel was not very pronounced.

### 3.3. Pellicle Ultrastructure

**Figure 5:**
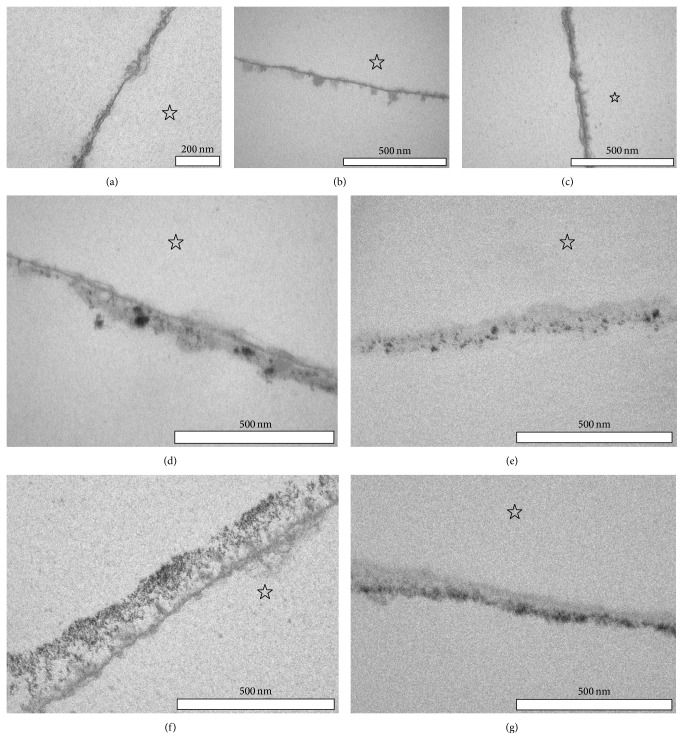
Transmission electron microscopic images of the* in situ* formed 30 min pellicle without pretreatment (a) and with pretreatment with Biorepair mouth rinsing solution (b, d, e) and GC Tooth Mousse (c, f, g) at 98.000-fold magnification. For representative images of the influence of elmex Kariesschutz on* in situ* pellicle formation previously published by our workgroup see Weber et al. [33]. The enamel was removed during the preparation of the samples; the former enamel site is marked with an asterisk. After 30 min of physiological pellicle formation a fine electron-dense basal layer (basal pellicle) as well as a thin fine granular second layer can be detected at the enamel surface (a). Clearly neither rinses with Biorepair mouth rinsing solution ((b, d) contrasted, (e) uncontrasted) nor the application of GC Tooth Mousse ((d, f) both contrasted, (g) uncontrasted) after 1 min of pellicle formation seemed to affect or penetrate the characteristic appearance of the basal layer. However, in 10% of the analyzed pellicle, ultrastructural changes that are possibly due to an accumulation of electron-dense mineral components could be visualized in the outer parts of the* in situ* formed pellicle 28 min after pretreatment with the prophylactic products (d–g). While the inorganic electron-dense aggregates after rinsing with Biorepair mouth rinsing solution appear randomly distributed throughout the pellicle layer (d, e), electron-dense inorganic particles are more homogenously arranged and densely attached to the outer parts of the* in situ* formed pellicle after application of the Tooth Mousse paste (f, g).

Selected samples of the physiological 30 min pellicle as well as the Biorepair mouth rinsing solution and GC Tooth Mousse-modified pellicle were investigated by TEM in order to identify possible ultrastructural differences (Figure 5). After 30 min of oral exposure, pellicle formation has occurred on all investigated enamel slabs. In line with several earlier investigations, the characteristic appearance of a continuous electron-dense basal layer as well as a more inhomogeneous and scattered second granular layer could be visualized, if no additional prophylactic measures were performed (Figure 5(a)). In the main, 1 min rinsing with Biorepair mouth rinsing solution or 1 min application of GC Tooth Mousse did not cause distinct changes in the ultrastructure of the 30 min* in situ* formed pellicle (Figures 5(b) and 5(c)). However, on 10% of the pellicle's length analyzed by TEM, an increase in pellicle thickness as well as incorporation of nanosized electron-dense particles in the pellicle layer was detectable after rinsing with the Biorepair mouth rinsing solution or application of the GC Tooth Mousse paste (Figures 5(d)–5(g)). In case of the Biorepair mouth rinsing solution, electron-dense particles were randomly distributed in the pellicle layer and varied in size from a few nanometers up to 100 nm (Figures 5(d) and 5(e)). In contrast, after application of the GC Tooth Mousse paste, the electron-dense particles revealed a size of only a few nanometers. These particles formed cluster-like aggregates and were predominantly enriched in the outer part of the pellicle (Figures 5(f) and 5(g)). The fact that these particles provided high electron density and were clearly detectable also in uncontrasted ultrathin sections indicates that they are composed of nonproteinaceous material.

## 4. Discussion

As one of very few, the present study investigated the impact of two different calcium phosphate and apatite containing preparations on* in situ* pellicle formation as well as their protective effect during erosive demineralization processes. Regarding the prevention of erosive surface destruction different approaches for the application of preventive agents are discussed. Numerous publications have described the morphological changes of dental hard tissue under erosive attack resulting from the collapse of demineralized crystallites [2, 6, 40]. The principally expected effect of modern micro- and nanoparticle based preparations is their precipitates to refill these defects and to smoothen the affected surface. Several studies have already addressed and confirmed the remineralizing potential of hydroxyapatite particles and CPP-ACP* in vitro* as well as* in vivo, *while the material's possible effects on preventing demineralization were given less consideration [23, 41–43]. Also, remembering that the imbalance of de- and remineralization is evidently influenced by the saliva and the components and charges within the pellicle layer, it is noteworthy that there is no data on the material's effect on the pellicle ultrastructure [1, 34, 44]. Furthermore, the formation of the pellicle layer* in situ* has generally been disregarded and most investigations were performed under rather experimental conditions such as overextended acid exposure times [17, 45].

As opposed to this, the present study investigated the efficacy of different calcium phosphate and apatite based preparations to enhance the protective properties of the* in situ* formed pellicle layer against erosive enamel demineralization. Since fluorides are generally recognized as the most common active ingredients in oral prophylactic preparations they served as a control. However, it has to be considered that, in terms of erosion prevention, the limited efficacy of purely sodium and amino fluoride based products has been discussed [1]. Furthermore, some of their effects on the dental tissue depend on a regular application. Therefore beneficial alternative fluoride preparations based on combined agents such as stannous fluoride have been suggested [1, 46–48]. Nevertheless, in the interest of comparability with previous studies and for the clarity of the question addressed in the present investigation, the sodium and amino fluoride based preparation was deliberately chosen as a control.

A predominant request of this study was a clinically relevant adaption of the investigated processes. In case of erosive oral conditions, the surfaces in the oral cavity are only exposed to acidic substances for a few seconds. Therefore, initial biocorrosive processes are focused on in particular in the present study. They are induced by the dissociation of calcium and phosphate from the outer enamel surface. In contrast to demineralization caused by lactic or bacterial acids, erosion associated defects of the dental tissue are usually caused by acids with a pH < 4.5, although the critical pH for tissue demineralization depends on several conditions [6]. The erosive potential of different common acidic solutions has been investigated in previous studies. Considering the etiology of dental erosion, hydrochloric acid qualifies as a suitable chemical to simulate acid exposure* in vitro*, as it is the major acid of intrinsic origin and it is closely related to several common pathologic conditions.

Although changes of the surface hardness evolve from the mineral extractions, the most specific determination of the mineral loss is achieved by photometric quantification of dissolved calcium and phosphate. A similar experimental approach was successfully used previously to evaluate the potential effect of rinses with edible oils or plant extracts on enamel erosion [33, 34].

Regarding the present investigation, the determination of calcium and phosphate release as the only method has its limitations because both hydroxyapatite microclusters and CPP-ACP are composed of the minerals. Consequently, a definite attribution on the origin of the measured ion-concentration-changes in the surrounding solution is not possible. Incubating the enamel slabs in HCl could have either resulted in demineralization of the enamel surface or in release of calcium and phosphate primarily from the modified pellicle, leaving the enamel surface rather intact [49]. It is conceivable that the formation of a particle based protective layer avoids the dissociation of ions out from the enamel but instead serves as an ion reservoir buffering the erosive attack. For this purpose, exemplary samples were additionally investigated by scanning electron microscopy in order to get an impression of the appearance of the enamel surface after erosive attack (Figure 4). In addition, valuable information could be derived from the transmission electron microscopic investigation of the* in situ* formed pellicle layer, as ultrastructural variations could be revealed for the different experimental samples (Figure 5) [34, 50]. The full comparability of the results has to be qualified by the fact that, depending on the individual product different application modes, mouthwash (elmex Kariesschutz, Biorepair mouth rinsing solution) or paste coating (GC Tooth Mousse) was performed. Nevertheless, we attempted adapting the experimental setup in accordance with the manufacturers' recommendations.

On the basis of the pellicles' analysis after 30 min formation time it can be suggested that all products initially ameliorated the protective properties of the pellicle layer. Thereby different physicochemical interactions of the products with the pellicle layer or the enamel surface might occur.

As expected, the application of fluorides enhanced the protection against demineralization. In an acidic environment, the hydroxyapatite structures of the enamel are dissolved but fluoride ions prevent extensive demineralization and enhance remineralization. The present findings confirm the efficacy of fluorides to a certain extent for the prevention of erosive mineral loss as shown electron-microscopically and especially via measurement of calcium and phosphate loss [1, 7]. Interestingly, considering the existing literature, little has so far been published about the possible interactions of fluorides with the* in situ* pellicle and the resulting impact on the pellicle's erosion-preventive potential, respectively. Evidence for pellicle modification by fluoride application has been obtained from quantitative proteomic analysis [4, 51]. While Siqueira et al. determined a decrease of statherin and histatin 1 after treatment with a 5% sodium fluoride solution [51], Algarni et al. measured an increase of MUC7, histatin 3, and acidic PRP after application of 0.025% sodium fluoride compared to the physiological pellicle [4]. However, all studies were solely performed* in vitro*. According to recent investigations, neither could mouthwashes with sodium and amino fluoride noticeably alter the ultrastructure of the* in situ* formed pellicle nor could a sustainable retention of fluoride at the dental tissue's surface be confirmed by EDX analysis [8, 33]. A previous study by O'Toole et al. investigated the effect of either stannous fluoride or sodium fluoride application before and after erosive attack with or without the presence of a salivary pellicle, however, under* in vitro* conditions [48]. While stannous fluoride appeared to be an effective substance in decreasing mineral loss, if applied before acid exposure, sodium fluoride was shown to be more appropriate for remineralizing the dental tissue after acid exposure.

As for the first time confirmed by transmission electron microscopy, GC Tooth Mousse might promote the accumulation of calcium and phosphate at the tooth surface under* in situ* conditions, since the casein phosphopeptide structures have a high affinity to the pellicle layer [49]. After application of GC Tooth Mousse in the present study, the pellicle layer formed* in situ* within 30 min partially appears much electron-denser than the 30 min control (Figure 5). Since the electron-dense particles were also detectable in the uncontrasted pellicle images, we conclude that they were of nonproteinaceous mineral content. However, no surface-wide modification of the pellicle by application of the GC Tooth Mousse paste was observed in the present study (Figure 5(c)). More precisely, incorporation of electron-dense particles in the pellicle layer was only detectable in approximately 10% of the analyzed pellicle length (Figures 5(f) and 5(g)). Based on these first TEM observations of the* in situ* pellicle, an impact of GC Tooth Mousse on the pellicle's ultrastructure can be assumed which, however, appears to be limited after 30 min of pellicle formation. Possible consequences of GC Tooth Mousse application include the following: the casein phosphopeptide molecules could reduce the permeability of the pellicle and enhance the tenacity of this proteinaceous layer. Furthermore, the accumulated calcium and phosphate ions might promote remineralization of the enamel structures and if acids interact with the GC Tooth Mousse-modified pellicle, the increased ion depot of the pellicle could serve as a reservoir. The present data and SEM-images support this hypothesis: after application of GC Tooth Mousse a reduced mineral loss was observed in most cases as compared with native enamel. However, there was still loss of calcium and phosphate, although the SEM-images showed a diminished surface destruction. This could indicate that calcium and phosphate obtained from GC Tooth Mousse application were dissolved first which suggests an effective protection against erosive attacks.

Also the hydroxyapatite microclusters contained in Biorepair mouth rinsing solution are assumed to form a layer on the enamel surface aggravating the process of demineralization or serving as reservoirs for enhanced remineralization. For the first time, TEM analysis confirmed a potential accumulation of hydroxyapatite microclusters in the outer parts of the 30 min pellicle due to the use of Biorepair mouth rinsing solution. However, again no continuous layer formation was observed in the present experiments (Figure 5(b)) and in contrast to GC Tooth Mousse application the SEM-images showed no delay of the erosive destruction. The precipitates of the microclusters appear occasionally distributed. First, this is probably due to the bigger size of the microclusters. In previous studies, the shape and size of the zinc-hydroxyapatite particles contained in the product used in this study have already been visualized by electron microscopy; crystallites of 15–20 nm appeared to aggregate to microclusters of 150–250 nm [3]. By contrast, the casein phosphopeptides stabilize amorphous calcium phosphate complexes with a diameter of 2.12 nm [52]. Furthermore a crucial structural feature of casein phosphopeptides might be their resemblance to pellicle-phosphopeptides which might enhance better integration into the complex pellicle structure [21, 49]. However, it must be considered that all observations are based on 30 min investigation time.

Also, in most cases the erosive calcium and phosphate loss after rinsing with Biorepair mouth rinsing solution were higher than after application of GC Tooth Mousse. This does not necessarily mean that hydroxyapatite microclusters have limited effect on dental erosion. Maybe higher concentrations are required; the tested product contained less than 1%. However, the present study only considered commercially available products.

## 5. Conclusion

The suitability of sodium and amino fluoride for preventing erosive damage to the hard dental tissue is limited and calcium phosphate and apatite based preparations are being investigated as potential biomimetic alternatives. The present study confirmed an efficacy of Biorepair mouth rinsing solution as well as GC Tooth Mousse to decrease acid-induced demineralization of the enamel surface, however, being inferior to fluoride application. Electron microscopic analysis indicates a pellicle modification by the individual products but the observed effects appeared rather randomly distributed than widespread throughout the pellicle layer.

## Figures and Tables

**Table 1:** Principle composition of prophylactic agents used in this study.

Prophylactic agent	Main ingredients	Percentage
Biorepair tooth and mouth rinsing solution (Dr. Wolff, Bielefeld, Germany)	Water	>50
	Sorbitol	<25
	Alcohol denat.	4
	Glycerin	<5
	Xylitol	<5
	Cellulose gum	<5
	Zinc hydroxyapatite	<1
	Zinc PCA, aroma, sodium lauryl sulfate, silica, *Ricinus communis* seed oil, ammonium acryloyldimethyltaurate/VP-copolymer, sodium saccharin, sodium benzoate	<1

GC Tooth Mousse (GC EUROPE N.V., Leuven, Belgium)	Water	>50
	Glycerol	10–25
	CPP-ACP	10
	CMC-Na	<5
	Silicon dioxide	<5
	Titanium dioxide	<5
	Zinc oxide	<5
	Propylene glycol, D-sorbitol, xylitol, phosphoric acid, flavoring, sodium saccharin, ethyl p-hydroxybenzoate, magnesium oxide, guar gum, propyl p-hydroxybenzoate, butyl p-hydroxybenzoate	

elmex Kariesschutz (GABA, Lörrach, Germany)	Water	>50
	150 ppm sodium fluoride	150 ppm
	100 ppm olaflur (amino fluoride)	100 ppm
	PEG-40 hydrogenated castor oil, aroma, propylene glycol, glycerin, sodium benzoate, levulinic acid, sodium levulinate, saccharin, sodium anisate	
